# Supplementary material for: HIVprotI: an integrated web based platform for prediction and design of HIV proteins inhibitors
Source: J Cheminform. 2018 Mar 9;10:12. doi: 10.1186/s13321-018-0266-y (PMC5845081; doi:10.1186/s13321-018-0266-y)
Supplement: Supplementary file 2 — Additional file 2. Source code of HIVProtI web server. [file 13321_2018_266_MOESM2_ESM.zip › HIVprotI_Source-code/hivprotI/weka-3-6-6/doc/weka/core/TestInstances.html]

TestInstances


---


|  |  |  |
| --- | --- | --- |
| **Overview** \| **Package** \| **Class** \| **Tree** \| **Deprecated** \| **Index** \| **Help** \| **Weka's home** | |  |
| **PREV CLASS**   **NEXT CLASS** | **FRAMES**    **NO FRAMES**     **All Classes** |  |
| SUMMARY: NESTED \| FIELD \| CONSTR \| METHOD | DETAIL: FIELD \| CONSTR \| METHOD |  |


---


## weka.core Class TestInstances

```
java.lang.Object
  weka.core.TestInstances
```

**All Implemented Interfaces:**: java.io.Serializable, java.lang.Cloneable, OptionHandler, RevisionHandler

---

``` public class TestInstances extends java.lang.Object implements java.lang.Cloneable, java.io.Serializable, OptionHandler, RevisionHandler ```

Generates artificial datasets for testing. In case of Multi-Instance data
the settings for the number of attributes applies to the data inside
the bag. Originally based on code from the CheckClassifier.


Valid options are:

```
 -relation <name>
  The name of the data set.
```

```
 -seed <num>
  The seed value.
```

```
 -num-instances <num>
  The number of instances in the datasets (default 20).
```

```
 -class-type <num>
  The class type, see constants in weka.core.Attribute
  (default 1=nominal).
```

```
 -class-values <num>
  The number of classes to generate (for nominal classes only)
  (default 2).
```

```
 -class-index <num>
  The class index, with -1=last, (default -1).
```

```
 -no-class
  Doesn't include a class attribute in the output.
```

```
 -nominal <num>
  The number of nominal attributes (default 1).
```

```
 -nominal-values <num>
  The number of values for nominal attributes (default 2).
```

```
 -numeric <num>
  The number of numeric attributes (default 0).
```

```
 -string <num>
  The number of string attributes (default 0).
```

```
 -words <comma-separated-list>
  The words to use in string attributes.
```

```
 -word-separators <chars>
  The word separators to use in string attributes.
```

```
 -date <num>
  The number of date attributes (default 0).
```

```
 -relational <num>
  The number of relational attributes (default 0).
```

```
 -relational-nominal <num>
  The number of nominal attributes in a rel. attribute (default 1).
```

```
 -relational-nominal-values <num>
  The number of values for nominal attributes in a rel. attribute (default 2).
```

```
 -relational-numeric <num>
  The number of numeric attributes in a rel. attribute (default 0).
```

```
 -relational-string <num>
  The number of string attributes in a rel. attribute (default 0).
```

```
 -relational-date <num>
  The number of date attributes in a rel. attribute (default 0).
```

```
 -num-instances-relational <num>
  The number of instances in relational/bag attributes (default 10).
```

```
 -multi-instance
  Generates multi-instance data.
```

```
 -W <classname>
  The Capabilities handler to base the dataset on.
  The other parameters can be used to override the ones
  determined from the handler. Additional parameters for
  handler can be passed on after the '--'.
```

**Version:**
:   $Revision: 6325 $

**Author:**
:   FracPete (fracpete at waikato dot ac dot nz)

**See Also:**: `CheckClassifier`, Serialized Form

---

| **Field Summary** | |
| --- | --- |
| `static int` | `CLASS_IS_LAST`             can be used for settting the class attribute index to last |
| `static java.lang.String` | `DEFAULT_SEPARATORS`             the default word separators used in strings |
| `static java.lang.String[]` | `DEFAULT_WORDS`             the default list of words used in strings |
| `static int` | `NO_CLASS`             can be used to avoid generating a class attribute |


**Constructor Summary**

`TestInstances()`
  
          the default constructor


| **Method Summary** | |
| --- | --- |
| `void` | `assign(TestInstances t)`             updates itself with all the settings from the given TestInstances object |
| `java.lang.Object` | `clone()`             creates a clone of the current object |
| `static TestInstances` | `forCapabilities(Capabilities c)`             returns a TestInstances instance setup already for the the given capabilities. |
| `Instances` | `generate()`             Generates a new dataset |
| `Instances` | `generate(java.lang.String namePrefix)`             generates a new dataset. |
| `int` | `getClassIndex()`             returns the current class index (0-based), -1 is last attribute |
| `int` | `getClassType()`             returns the current class type |
| `Instances` | `getData()`             returns the current dataset, can be null |
| `CapabilitiesHandler` | `getHandler()`             returns the current set CapabilitiesHandler to generate the dataset for, can be null |
| `boolean` | `getMultiInstance()`             Gets whether multi-instance data (with a fixed structure) is generated |
| `boolean` | `getNoClass()`             whether no class attribute is generated |
| `int` | `getNumAttributes()`             returns the overall number of attributes (incl. |
| `int` | `getNumClasses()`             returns the current number of classes |
| `int` | `getNumDate()`             returns the current number of date attributes |
| `int` | `getNumInstances()`             returns the current number of instances to produce |
| `int` | `getNumInstancesRelational()`             returns the current number of instances in relational/bag attributes to produce |
| `int` | `getNumNominal()`             returns the current number of nominal attributes |
| `int` | `getNumNominalValues()`             returns the current number of values for nominal attributes |
| `int` | `getNumNumeric()`             returns the current number of numeric attributes |
| `int` | `getNumRelational()`             returns the current number of relational attributes |
| `int` | `getNumRelationalDate()`             returns the current number of date attributes in a relational attribute |
| `int` | `getNumRelationalNominal()`             returns the current number of nominal attributes in a relational attribute |
| `int` | `getNumRelationalNominalValues()`             returns the current number of values for nominal attributes in a relational attribute |
| `int` | `getNumRelationalNumeric()`             returns the current number of numeric attributes in a relational attribute |
| `int` | `getNumRelationalString()`             returns the current number of string attributes in a relational attribute |
| `int` | `getNumString()`             returns the current number of string attributes |
| `java.lang.String[]` | `getOptions()`             Gets the current settings of this object. |
| `java.lang.String` | `getRelation()`             returns the current name of the relation |
| `Instances` | `getRelationalClassFormat()`             returns the current strcuture of the relational class attribute, can be null |
| `Instances` | `getRelationalFormat(int index)`             returns the format for the specified relational attribute, can be null |
| `java.lang.String` | `getRevision()`             Returns the revision string. |
| `int` | `getSeed()`             returns the current seed value |
| `java.lang.String` | `getWords()`             returns the words used for assembling strings in a comma-separated list. |
| `java.lang.String` | `getWordSeparators()`             returns the word separators (chars) to use for assembling strings. |
| `java.util.Enumeration` | `listOptions()`             Returns an enumeration describing the available options. |
| `static void` | `main(java.lang.String[] args)`             for running the class from commandline, prints the generated data to stdout |
| `void` | `setClassIndex(int value)`             sets the class index (0-based) |
| `void` | `setClassType(int value)`             sets the class attribute type |
| `void` | `setHandler(CapabilitiesHandler value)`             sets the Capabilities handler to generate the data for |
| `void` | `setMultiInstance(boolean value)`             sets whether multi-instance data should be generated (with a fixed data structure) |
| `void` | `setNoClass(boolean value)`             whether to have no class, e.g., for clusterers; otherwise the class attribute index is set to last |
| `void` | `setNumClasses(int value)`             sets the number of classes |
| `void` | `setNumDate(int value)`             sets the number of date attributes |
| `void` | `setNumInstances(int value)`             sets the number of instances to produce |
| `void` | `setNumInstancesRelational(int value)`             sets the number of instances in relational/bag attributes to produce |
| `void` | `setNumNominal(int value)`             sets the number of nominal attributes |
| `void` | `setNumNominalValues(int value)`             sets the number of values for nominal attributes |
| `void` | `setNumNumeric(int value)`             sets the number of numeric attributes |
| `void` | `setNumRelational(int value)`             sets the number of relational attributes |
| `void` | `setNumRelationalDate(int value)`             sets the number of date attributes in a relational attribute |
| `void` | `setNumRelationalNominal(int value)`             sets the number of nominal attributes in a relational attribute |
| `void` | `setNumRelationalNominalValues(int value)`             sets the number of values for nominal attributes in a relational attribute |
| `void` | `setNumRelationalNumeric(int value)`             sets the number of numeric attributes in a relational attribute |
| `void` | `setNumRelationalString(int value)`             sets the number of string attributes in a relational attribute |
| `void` | `setNumString(int value)`             sets the number of string attributes |
| `void` | `setOptions(java.lang.String[] options)`             Parses a given list of options. |
| `void` | `setRelation(java.lang.String value)`             sets the name of the relation |
| `void` | `setRelationalClassFormat(Instances value)`             sets the structure for the relational class attribute |
| `void` | `setRelationalFormat(int index, Instances value)`             sets the structure for the bags for the relational attribute |
| `void` | `setSeed(int value)`             sets the seed value for the random number generator |
| `void` | `setWords(java.lang.String value)`             Sets the comma-separated list of words to use for generating strings. |
| `void` | `setWordSeparators(java.lang.String value)`             sets the word separators (chars) to use for assembling strings. |
| `java.lang.String` | `toString()`             returns a string representation of the object |

**Methods inherited from class java.lang.Object**

`equals, getClass, hashCode, notify, notifyAll, wait, wait, wait`

**Field Detail**

### CLASS\_IS\_LAST

```
public static final int CLASS_IS_LAST
```

:   can be used for settting the class attribute index to last

    **See Also:**: `setClassIndex(int)`, Constant Field Values

---


### NO\_CLASS

```
public static final int NO_CLASS
```

:   can be used to avoid generating a class attribute

    **See Also:**: `setClassIndex(int)`, Constant Field Values

---


### DEFAULT\_WORDS

```
public static final java.lang.String[] DEFAULT_WORDS
```

:   the default list of words used in strings

---


### DEFAULT\_SEPARATORS

```
public static final java.lang.String DEFAULT_SEPARATORS
```

:   the default word separators used in strings

    **See Also:**: Constant Field Values


**Constructor Detail**

### TestInstances

```
public TestInstances()
```

:   the default constructor


**Method Detail**

### clone

```
public java.lang.Object clone()
```

:   creates a clone of the current object

    :   **Overrides:**: `clone` in class `java.lang.Object`
    :   **Returns:**: a clone of the current object

---


### assign

```
public void assign(TestInstances t)
```

:   updates itself with all the settings from the given TestInstances
    object

    :   **Parameters:**: `t` - the object to get the settings from

---


### listOptions

```
public java.util.Enumeration listOptions()
```

:   Returns an enumeration describing the available options.

    :   **Specified by:**: `listOptions` in interface `OptionHandler`
    :   **Returns:**: an enumeration of all the available options.

---


### setOptions

```
public void setOptions(java.lang.String[] options)
                throws java.lang.Exception
```

:   Parses a given list of options.


    Valid options are:

    ```
     -relation <name>
      The name of the data set.
    ```

    ```
     -seed <num>
      The seed value.
    ```

    ```
     -num-instances <num>
      The number of instances in the datasets (default 20).
    ```

    ```
     -class-type <num>
      The class type, see constants in weka.core.Attribute
      (default 1=nominal).
    ```

    ```
     -class-values <num>
      The number of classes to generate (for nominal classes only)
      (default 2).
    ```

    ```
     -class-index <num>
      The class index, with -1=last, (default -1).
    ```

    ```
     -no-class
      Doesn't include a class attribute in the output.
    ```

    ```
     -nominal <num>
      The number of nominal attributes (default 1).
    ```

    ```
     -nominal-values <num>
      The number of values for nominal attributes (default 2).
    ```

    ```
     -numeric <num>
      The number of numeric attributes (default 0).
    ```

    ```
     -string <num>
      The number of string attributes (default 0).
    ```

    ```
     -words <comma-separated-list>
      The words to use in string attributes.
    ```

    ```
     -word-separators <chars>
      The word separators to use in string attributes.
    ```

    ```
     -date <num>
      The number of date attributes (default 0).
    ```

    ```
     -relational <num>
      The number of relational attributes (default 0).
    ```

    ```
     -relational-nominal <num>
      The number of nominal attributes in a rel. attribute (default 1).
    ```

    ```
     -relational-nominal-values <num>
      The number of values for nominal attributes in a rel. attribute (default 2).
    ```

    ```
     -relational-numeric <num>
      The number of numeric attributes in a rel. attribute (default 0).
    ```

    ```
     -relational-string <num>
      The number of string attributes in a rel. attribute (default 0).
    ```

    ```
     -relational-date <num>
      The number of date attributes in a rel. attribute (default 0).
    ```

    ```
     -num-instances-relational <num>
      The number of instances in relational/bag attributes (default 10).
    ```

    ```
     -multi-instance
      Generates multi-instance data.
    ```

    ```
     -W <classname>
      The Capabilities handler to base the dataset on.
      The other parameters can be used to override the ones
      determined from the handler. Additional parameters for
      handler can be passed on after the '--'.
    ```

    :   **Specified by:**: `setOptions` in interface `OptionHandler`
    :   **Parameters:**: `options` - the list of options as an array of strings **Throws:**: `java.lang.Exception` - if an option is not supported

---


### getOptions

```
public java.lang.String[] getOptions()
```

:   Gets the current settings of this object.

    :   **Specified by:**: `getOptions` in interface `OptionHandler`
    :   **Returns:**: an array of strings suitable for passing to setOptions

---


### setRelation

```
public void setRelation(java.lang.String value)
```

:   sets the name of the relation

    :   **Parameters:**: `value` - the name of the relation

---


### getRelation

```
public java.lang.String getRelation()
```

:   returns the current name of the relation

    :   **Returns:**: the name of the relation

---


### setSeed

```
public void setSeed(int value)
```

:   sets the seed value for the random number generator

    :   **Parameters:**: `value` - the seed

---


### getSeed

```
public int getSeed()
```

:   returns the current seed value

    :   **Returns:**: the seed value

---


### setNumInstances

```
public void setNumInstances(int value)
```

:   sets the number of instances to produce

    :   **Parameters:**: `value` - the number of instances

---


### getNumInstances

```
public int getNumInstances()
```

:   returns the current number of instances to produce

    :   **Returns:**: the number of instances

---


### setClassType

```
public void setClassType(int value)
```

:   sets the class attribute type

    :   **Parameters:**: `value` - the class attribute type

---


### getClassType

```
public int getClassType()
```

:   returns the current class type

    :   **Returns:**: the class attribute type

---


### setNumClasses

```
public void setNumClasses(int value)
```

:   sets the number of classes

    :   **Parameters:**: `value` - the number of classes

---


### getNumClasses

```
public int getNumClasses()
```

:   returns the current number of classes

    :   **Returns:**: the number of classes

---


### setClassIndex

```
public void setClassIndex(int value)
```

:   sets the class index (0-based)

    :   **Parameters:**: `value` - the class index **See Also:**: `CLASS_IS_LAST`, `NO_CLASS`

---


### getClassIndex

```
public int getClassIndex()
```

:   returns the current class index (0-based), -1 is last attribute

    :   **Returns:**: the class index **See Also:**: `CLASS_IS_LAST`, `NO_CLASS`

---


### setNoClass

```
public void setNoClass(boolean value)
```

:   whether to have no class, e.g., for clusterers; otherwise the class
    attribute index is set to last

    :   **Parameters:**: `value` - whether to have no class **See Also:**: `CLASS_IS_LAST`, `NO_CLASS`

---


### getNoClass

```
public boolean getNoClass()
```

:   whether no class attribute is generated

    :   **Returns:**: true if no class attribute is generated

---


### setNumNominal

```
public void setNumNominal(int value)
```

:   sets the number of nominal attributes

    :   **Parameters:**: `value` - the number of nominal attributes

---


### getNumNominal

```
public int getNumNominal()
```

:   returns the current number of nominal attributes

    :   **Returns:**: the number of nominal attributes

---


### setNumNominalValues

```
public void setNumNominalValues(int value)
```

:   sets the number of values for nominal attributes

    :   **Parameters:**: `value` - the number of values

---


### getNumNominalValues

```
public int getNumNominalValues()
```

:   returns the current number of values for nominal attributes

    :   **Returns:**: the number of values

---


### setNumNumeric

```
public void setNumNumeric(int value)
```

:   sets the number of numeric attributes

    :   **Parameters:**: `value` - the number of numeric attributes

---


### getNumNumeric

```
public int getNumNumeric()
```

:   returns the current number of numeric attributes

    :   **Returns:**: the number of numeric attributes

---


### setNumString

```
public void setNumString(int value)
```

:   sets the number of string attributes

    :   **Parameters:**: `value` - the number of string attributes

---


### getNumString

```
public int getNumString()
```

:   returns the current number of string attributes

    :   **Returns:**: the number of string attributes

---


### setWords

```
public void setWords(java.lang.String value)
```

:   Sets the comma-separated list of words to use for generating strings. The
    list must contain at least 2 words, otherwise an exception will be thrown.

    :   **Parameters:**: `value` - the list of words **Throws:**: `java.lang.IllegalArgumentException` - if not at least 2 words are provided

---


### getWords

```
public java.lang.String getWords()
```

:   returns the words used for assembling strings in a comma-separated list.

    :   **Returns:**: the words as comma-separated list

---


### setWordSeparators

```
public void setWordSeparators(java.lang.String value)
```

:   sets the word separators (chars) to use for assembling strings.

    :   **Parameters:**: `value` - the characters to use as separators

---


### getWordSeparators

```
public java.lang.String getWordSeparators()
```

:   returns the word separators (chars) to use for assembling strings.

    :   **Returns:**: the current separators

---


### setNumDate

```
public void setNumDate(int value)
```

:   sets the number of date attributes

    :   **Parameters:**: `value` - the number of date attributes

---


### getNumDate

```
public int getNumDate()
```

:   returns the current number of date attributes

    :   **Returns:**: the number of date attributes

---


### setNumRelational

```
public void setNumRelational(int value)
```

:   sets the number of relational attributes

    :   **Parameters:**: `value` - the number of relational attributes

---


### getNumRelational

```
public int getNumRelational()
```

:   returns the current number of relational attributes

    :   **Returns:**: the number of relational attributes

---


### setNumRelationalNominal

```
public void setNumRelationalNominal(int value)
```

:   sets the number of nominal attributes in a relational attribute

    :   **Parameters:**: `value` - the number of nominal attributes

---


### getNumRelationalNominal

```
public int getNumRelationalNominal()
```

:   returns the current number of nominal attributes in a relational attribute

    :   **Returns:**: the number of nominal attributes

---


### setNumRelationalNominalValues

```
public void setNumRelationalNominalValues(int value)
```

:   sets the number of values for nominal attributes in a relational attribute

    :   **Parameters:**: `value` - the number of values

---


### getNumRelationalNominalValues

```
public int getNumRelationalNominalValues()
```

:   returns the current number of values for nominal attributes in a relational attribute

    :   **Returns:**: the number of values

---


### setNumRelationalNumeric

```
public void setNumRelationalNumeric(int value)
```

:   sets the number of numeric attributes in a relational attribute

    :   **Parameters:**: `value` - the number of numeric attributes

---


### getNumRelationalNumeric

```
public int getNumRelationalNumeric()
```

:   returns the current number of numeric attributes in a relational attribute

    :   **Returns:**: the number of numeric attributes

---


### setNumRelationalString

```
public void setNumRelationalString(int value)
```

:   sets the number of string attributes in a relational attribute

    :   **Parameters:**: `value` - the number of string attributes

---


### getNumRelationalString

```
public int getNumRelationalString()
```

:   returns the current number of string attributes in a relational attribute

    :   **Returns:**: the number of string attributes

---


### setNumRelationalDate

```
public void setNumRelationalDate(int value)
```

:   sets the number of date attributes in a relational attribute

    :   **Parameters:**: `value` - the number of date attributes

---


### getNumRelationalDate

```
public int getNumRelationalDate()
```

:   returns the current number of date attributes in a relational attribute

    :   **Returns:**: the number of date attributes

---


### setNumInstancesRelational

```
public void setNumInstancesRelational(int value)
```

:   sets the number of instances in relational/bag attributes to produce

    :   **Parameters:**: `value` - the number of instances

---


### getNumInstancesRelational

```
public int getNumInstancesRelational()
```

:   returns the current number of instances in relational/bag attributes to produce

    :   **Returns:**: the number of instances

---


### setMultiInstance

```
public void setMultiInstance(boolean value)
```

:   sets whether multi-instance data should be generated (with a fixed
    data structure)

    :   **Parameters:**: `value` - whether multi-instance data is generated

---


### getMultiInstance

```
public boolean getMultiInstance()
```

:   Gets whether multi-instance data (with a fixed structure) is generated

    :   **Returns:**: true if multi-instance data is generated

---


### setRelationalFormat

```
public void setRelationalFormat(int index,
                                Instances value)
```

:   sets the structure for the bags for the relational attribute

    :   **Parameters:**: `index` - the index of the relational attribute: `value` - the new structure

---


### getRelationalFormat

```
public Instances getRelationalFormat(int index)
```

:   returns the format for the specified relational attribute, can be null

    :   **Parameters:**: `index` - the index of the relational attribute **Returns:**: the current structure

---


### setRelationalClassFormat

```
public void setRelationalClassFormat(Instances value)
```

:   sets the structure for the relational class attribute

    :   **Parameters:**: `value` - the structure for the relational attribute

---


### getRelationalClassFormat

```
public Instances getRelationalClassFormat()
```

:   returns the current strcuture of the relational class attribute, can
    be null

    :   **Returns:**: the relational structure of the class attribute

---


### getNumAttributes

```
public int getNumAttributes()
```

:   returns the overall number of attributes (incl. class, if that is also
    generated)

    :   **Returns:**: the overall number of attributes

---


### getData

```
public Instances getData()
```

:   returns the current dataset, can be null

    :   **Returns:**: the current dataset

---


### setHandler

```
public void setHandler(CapabilitiesHandler value)
```

:   sets the Capabilities handler to generate the data for

    :   **Parameters:**: `value` - the handler to generate the data for

---


### getHandler

```
public CapabilitiesHandler getHandler()
```

:   returns the current set CapabilitiesHandler to generate the dataset
    for, can be null

    :   **Returns:**: the handler to generate the data for

---


### generate

```
public Instances generate()
                   throws java.lang.Exception
```

:   Generates a new dataset

    :   **Returns:**: the generated data **Throws:**: `java.lang.Exception` - if something goes wrong

---


### generate

```
public Instances generate(java.lang.String namePrefix)
                   throws java.lang.Exception
```

:   generates a new dataset.

    :   **Parameters:**: `namePrefix` - the prefix to add to the name of an attribute **Returns:**: the generated data **Throws:**: `java.lang.Exception` - if something goes wrong

---


### forCapabilities

```
public static TestInstances forCapabilities(Capabilities c)
```

:   returns a TestInstances instance setup already for the the given
    capabilities.

    :   **Parameters:**: `c` - the capabilities to base the TestInstances on **Returns:**: the configured TestInstances object

---


### toString

```
public java.lang.String toString()
```

:   returns a string representation of the object

    :   **Overrides:**: `toString` in class `java.lang.Object`
    :   **Returns:**: a string representation of the object

---


### getRevision

```
public java.lang.String getRevision()
```

:   Returns the revision string.

    :   **Specified by:**: `getRevision` in interface `RevisionHandler`
    :   **Returns:**: the revision

---


### main

```
public static void main(java.lang.String[] args)
                 throws java.lang.Exception
```

:   for running the class from commandline, prints the generated data
    to stdout

    :   **Parameters:**: `args` - the commandline parameters **Throws:**: `java.lang.Exception` - if something goes wrong


---


|  |  |  |
| --- | --- | --- |
| **Overview** \| **Package** \| **Class** \| **Tree** \| **Deprecated** \| **Index** \| **Help** \| **Weka's home** | |  |
| **PREV CLASS**   **NEXT CLASS** | **FRAMES**    **NO FRAMES**     **All Classes** |  |
| SUMMARY: NESTED \| FIELD \| CONSTR \| METHOD | DETAIL: FIELD \| CONSTR \| METHOD |  |


---
